# Laparotomy

**Published:** 1878-11

**Authors:** W. S. Caldwell

**Affiliations:** Paris


No. II.

### LAPAROTOMY.—SUCCESSFUL EXTIRPATION OF FLOATING KIDNEY.

In visiting the surgical wards of the hospitals of Europe, and contrasting what I now see, with what I saw when abroad twelve years ago, there is no procedure in surgery that strikes me as having made so rapid a stride in that time as laparotomy.

An operation that a quarter of a century ago was considered fraught with the greatest danger, is now resorted to by many of the operators on the continent, with apparently as little hesitation as that with which the ordinary surgeon will open an abscess.

Soon after I came to Berlin this summer, Prof. Schröder brought a woman before the class who had a large abdominal swelling which he thought was an ovarian fibroid tumor; but not being well satisfied as to the diagnosis he proposed to make an abdominal section, in order to clear up this point, as well as to see what could be done in the way of relief by the knife.

He made an incision in the median line extending from near the symphysis pubis to the umbilicus, brought the tumor well into view, in fact brought with it a large portion of the small intestines entirely without the abdominal walls.

The growth proved to be a cancer of the mesenteric glands, and so attached that its removal was out of the question.

The wound was closed up and dressed with Prof. Lister's dressing—the operation having been done under the carbolized spray—and the patient recovered without an untoward symptom. In a paper on ovariotomy lately read by Dr. Martin, before the *Berliner Medicinische Gesellschaft*, he took strong grounds in favor of the almost absolute safety of this operation when properly and antiseptically done.

During the discussion of this paper, Dr. Küster related that he had lately opened the abdomen freely, to clear up the diagnosis in a case that proved to be one of carcinoma of the left ovary, having extensive attachment with the peritoneum. Removal could not be effected.

The wound was closed up, healed by first intention, and, at the end of eight days, the patient was as well as usual.

When she afterwards died from the effects of the cancer, the autopsy showed that the effect of this grave operation had left no traces behind it, save the slightest remains of a circumscribed peritonitis in the median line.

The speaker (Dr. K.) believed that in case of abdominal growths, where it was impossible to arrive at a correct diagnosis by other means, that laparotomy might be resorted to with absolute safety.

This discussion brought up another question of great importance to the ovariotomist, which is that of the carbolic acid poisoning said to sometimes follow the use of the carbolized spray.

Dr. Martin was of the opinion that the dangers from this accident were greatly over-estimated, while Dr. Küster believed that in case of children or very anæmic persons of adult age, that a solution of from 2 to 2½ per cent., used as a spray, was very dangerous.

The latter gentleman related a case of ovariotomy that he had lately done, under a spray of thymic acid, the patient having recovered without any bad symptoms, and he strongly urged this agent as a substitute for the carbolic acid. He used it of a strength of 1-10th per cent., and considers it as absolutely free from any danger of a toxic character, as well as being equal to the carbolic acid as an antiseptic agent.

During the past winter I saw Prof. Billroth perform ovariotomy under the spray; the patient, soon after the operation, passed into a comatose condition, and died, as Prof. B. thought, from carbolic acid intoxication.

After that the room (a small one) in which these operations were done, was thoroughly impregnated with the carbolized spray, but none was used at the time of the operation itself.

On the other hand Prof. Shröder, of Berlin, does all his ovariotomies under a 2 per cent. carbolized spray with the most happy results, and has no fears of any injurious influence from this agent.

Since last February, Prof. S. has done this operation forty-five times with only four fatal results, and of his last thirty patients he has not lost a single one. In performing ovariotomy Prof. Shröder uses no clamp, and dispenses with all drainage.

The cases that I saw him operate upon, were simple ones, with no very great extent of adhesion. He tied the pedicle with a silk ligature and closed up the abdominal walls completely. I asked him if in case of extensive adhesions and considerable hæmorrhage he would not adopt some form of drainage. His reply was that in his early operations it was his custom to use some method of drainage, but that latterly he had used none, and since this change his results had been much more favorable.

Billroth always used, in the cases I saw him operate upon, the ordinary drainage tube draining through the abdominal walls dropping the lower end of the tube well down into the cul de sac of Douglas. His statistics I believe are not good as compared with those of other prominent ovariotomists.

I saw Prof. Shröder extirpate the entire uterus in a woman, 42 years of age, for cancer of that organ. The patient made a good recovery, though not without considerable constitutional disturbance. This was his third operation of the kind, only one of the cases proving fatal.

I assisted Dr. Martin in the performance of this same operation on a rather delicate woman of 47 years of age. In her case the pelvic lymphatics were found considerably involved, and, in the doctor's efforts at their removal, the patient lost considerable blood and never reacted well after the operation, dying 18 hours after it was performed.

Extirpation of the Right Kidney.—On the morning of July 25th I assisted Dr. Martin in the performance of the operation named above.

The patient was an unmarried woman, aged 29 years, and had one child. For the last two years she had suffered most intensely from a pain in the right hypochondrium, extending around the entire diaphragm and down as far as the right iliac fossa.

The patient, although apparently in moderate health, had scarcely as she asserted, been free from pain for the last year.

Physical examination showed her to be suffering from a floating kidney; and although the organ was far less movable than in many of the cases I saw in the private course of Dr. Oser, of Vienna, her sufferings were all attributed to this cause, and hence this very grave operation was resorted to.

An incision was made in the median line, extending from near the ensiform cartilage to the umbilicus. After this free incision was made, it was found that the kidney had fallen so far back into its natural position that it could not be reached until the patient had been turned nearly on to her abdomen.

The organ was now caught with a strong pair of rat-tooth forceps, brought forward toward the opening in the abdominal walls, and its peritoneal covering divided by the knife.

The vessels that supply it were next ligated by three strong silk ligatures applied about three centimeters distant from the kidney itself, leaving a long flat pedicle after the organ was cut away.

The hemorrhage that followed the operation was considerable, but great pains was taken to remove all blood from the abdominal cavity by means of sponges wrung from a 2 per cent. solution of carbolic acid.

The wound was closed in the usual manner and dressed antiseptically, the operation having been done, of course, under the spray.

The patient was now placed in a well-ventilated and isolated room, kept perfectly quiet, not being allowed to even raise upright to pass her urine.

To give some idea of the constitutional disturbance that followed the operation, I append the following notes of her temperature:

July 25th, 8 o'clock a. m., temp., 36.4°. On the following days the temperature ranged thus: 26th, from 37.3° to 38°; 27th, from 37.3° to 38.7°; 28th, 38° to 39°; 29th, 37.5° to 38.6°; 30th, from 37.9° to 38.3°; 31st, from 37° to 38°; August 1st, from 36.6° to 37.5°.

I watched the patient for a month, during the first three weeks of which time she had every day a slight elevation of temperature.

When I last saw her she was nearly convalescent. The urinary secretion was about two-thirds of the normal amount for the first three weeks, and near four-fifths after that, and never was markedly altered in its chemical constituents.

This is Dr. Martin's third operation of this kind, and, wonderful to relate, his cases have all recovered.

Professor Billroth (I believe) has done the operation the same number of times but his cases have all died, though I think they were all performed for malignant or other diseases of the kidney. Professor B.'s mode of operating is to make an incision through the lumbar region, reaching the organ with less violence, as he thinks, to the peritoneum and adjacent tissues.

Dr. Oser claims that one woman in ten, who have borne children, have a movable right kidney, which he attributes to the loose manner in which this organ is bound down by its peritoneal attachments on this side, the ascent of the uterus during pregnancy loosening up these attachments.

He further claims that this affection plays an important role in the nervous diseases of females, which is often overlooked by those who are not accustomed to the more minute methods of physical exploration. W. S. Caldwell.

Paris, Sept. 20th.

